# Investigative Interviewing Research: Ideas and Methodological Suggestions for New Research Perspectives

**DOI:** 10.3389/fpsyg.2021.715028

**Published:** 2021-07-15

**Authors:** Nicola Palena, Letizia Caso

**Affiliations:** ^1^Department of Human and Social Sciences, University of Bergamo, Bergamo, Italy; ^2^Department of Human Sciences, Lumsa University of Rome, Rome, Italy

**Keywords:** investigative interviewing, lie detection, overlapping measure, person-centred analyses, intersubjectivity

## Introduction

Research studies concerning investigative interviewing and lie detection have seen a steep rise in recent years. Detecting lying is a human ambition and an interesting research question. Yet, people often hold wrong stereotypes about how to detect lies (The Global Deception Research Team, 2006), which makes paramount sound research studies. Two examples are the use of non-verbal communication and microexpressions as a means for lie detection. Neither of the two showed to be a reliable approach to detect lies (DePaulo et al., 2003; Burgoon, 2018; Jordan et al., 2019), and both have been criticised as ineffective (for two recent overviews, see Vrij et al., 2019; Brennen and Magnussen, 2020). Recent research studies prioritise verbal content (Masip et al., 2005), which appears to be a better tool for credibility assessment (Vrij, 2015; Amado et al., 2016) and on developing interviewing approaches that aim at enhancing the differences between truth tellers and liars (Vrij and Granhag, 2012, 2014). Yet, we are far from being able to accurately and reliably discriminate truth tellers from liars. The reasons can be traced back to several issues.

## The Issues

There are several issues related to lie detection. First, a unified theory of lying is lacking (Vrij, 2008; Bond et al., 2015; Nahari et al., 2019), which hence needs to be developed accounting for several factors together (e.g., cognitive, social, neurological and strategical decision-making processes, and linguistics). Second, people are good liars but bad judges (Bond and DePaulo, 2006; Levine, 2010). Third, different people show different cues to deception. Fourth, we have generally overlooked the importance of intersubjectivity. An exception is the Interpersonal Deception Theory by Buller and Burgoon (2006), which stresses the importance of the interaction between the sender and the receiver, but this theory has proven controversial (Bond et al., 2015). Fifth, deception detection should not be seen as an endpoint; rather, it should go in parallel with eliciting information (Gränhag, in Nahari et al., 2019).

Other issues are related to the methodological and analytic approaches that have been usually employed. We overlooked several measures, strategies and statistical analyses that can be useful to face some of the issues listed above.

## The Proposals

### Account for Interpersonal Differences and Intersubjectivity

One interviewing technique that aims to limit the effect of interpersonal differences is the baseline approach, which predicts that if an observer has a truthful baseline of the sender, it would be easier to detect deception. Yet, this approach appears controversial and mostly ineffective (Vrij, 2016; Caso et al.,2019a,b). Efforts have been made to improve its efficacy (Palena et al., 2019; Verigin et al., 2020; Tomas et al., 2021b), and a recent study has questioned its relevance, without rejecting it either (Tomas et al., 2021a). Furthermore, although the baseline attempts to deal with personal characteristics, almost all research studies in investigative interviewing used a variable-centred approach (Magnusson, 1992, 1998). This might be problematic as such an approach lays on the assumption that an effect (the relationship between several variables) is the same for the entire population and describes it with a single set of parameters. Nothing is told about the effect of personal characteristics. Although the variable-centred approach is parsimonious (results are easy to interpret), it has low specificity (low precision in describing a specific subject or subgroup).

A different approach with increasing popularity is the person-centred approach, which allows for the study of people in an integrative manner (Magnusson, 1998). In this study, the experimenter starts by selecting the variables of interest, which will then be used to group people into specific subpopulations, often called “profiles,” *via* mixture models of cluster analyses. People within a particular profile will be more similar in the patterns of scores on the selected variables than people belonging to a different profile. Once such profiles are obtained, they can then be put in a relationship with other predictors/outcome variables. The main point is that in the variable-centred approach it is presumed that an effect is the same across individuals, whereas in the person-centred approach it is thought that an effect can be different among people. To give an example, an experimenter adopting the variable-centred approach may look at the effect of veracity (truth tellers vs. liars) on the amount of details provided. On the contrary, a researcher adopting the person-centred approach may predict that such an effect is not the same for everyone. Hence, the researcher will explore how different personality profiles (e.g., one profile with high extraversion and high neuroticism vs. one profile with high extraversion but low neuroticism) moderate the relationship between veracity (the predictor) and the outcome (e.g., the amount of details) (Palena et al., under review). It follows that unlike the variable-centred approach, the person-centred approach (i) may provide several sets of parameters; (ii) is more specific but less parsimonious than the variable-centred approach; and (iii) can deal with the issue of interpersonal differences/personal characteristics better than the variable-centred approach. A possible difficulty for adopting this approach is that it requires larger samples of participants (usually > 500, Meyer and Morin, 2016) than those usually used in investigative interviewing research studies (for a detailed overview of the person-specific approach, see Magnusson, 1998). There are also specific approaches such as latent transition analysis that can also explore how people move from one profile to another over time (Lanza et al., 2010), which can be particularly useful to determine what intervening variables may push high-value interviewees moving from a profile indicating uncooperativeness to one indicating cooperativeness.

The last example is the person-specific approach, which aims to explore the effects that are specific to each individual. In this study, scores of the variables of interest are collected on many occasions and inferences are usually made on the individual rather than the profile. It follows that the person-specific approach is the more specific but the least parsimonious of the three.

The person-centred and the person-specific approaches deal with interpersonal characteristics better than the variable-centred approach, but they are still missing the role of the relationship that is built between the interviewer and the interviewee. This can be studied *via* dyadic analysis such as the actor-partner interdependence model (Cook and Kenny, 2005), which deals with the influence that one individual of the dyad has on the other individual and vice versa. Being investigative interviewing an interactive process between the interviewer(s) and the interviewee(s), such models become relevant.

### Methodological and Analytic Suggestions

Investigative interviewing research studies also suffer from methodological and analytical issues. One is related to the reporting of effect sizes. Most of the time, researchers report standardised mean differences, usually Cohen’s *d*, which is not easily interpretable and tells nothing about the efficacy of an interviewing technique or a deception cue to discriminate truth tellers from liars. Imagine a *d* = 0.50 with a hypothesis suggesting that truth tellers obtain a higher score than liars on a specific variable. Although this is a “medium” effect, the use of additional statistics might shed light on its true usefulness.

One approach is to explore the overlap between the distributions among the groups. Cohen (1988), for example, developed the *U*_3_ statistics, which expresses the percentages of scores in the group with the lower mean that are exceeded by the mean of the group with the higher score. The Probability of Superiority is instead the probability that a person taken randomly from one group will have a higher score than a person picked randomly from the other group. With a Cohen’s *d* = 0.50, 80.30% of the two groups (e.g., truth tellers and liars) will still overlap, the U3 would be of 69.15%, and the Probability of Superiority would be of 63.80%. Hence, the discriminability here is, in fact, very limited. Other useful measures are the Probability of Superiority of Effect Sizes (PSES), which transforms the effect sizes as percentiles (Arce et al., 2015; Monteiro et al., 2018), the Probability of Inferiority Score (PIS), which is the probability that one group obtains a score that is lower than the mean score of another group (Monteiro et al., 2018; Arias et al., 2020), and the discriminant coefficient (DISCO), which describes the proportion of people in one group that fall below the lower score of the other group (Guttman, 1988, 1989). The problem is that measures such as Cohen’s *U*_3_ require specific proprieties of the distribution(s), such as unimodality and symmetry. A recent approach is very useful in dealing with this issue as it is a distribution-free overlapping measure (Pastore and Calcagnì, 2019), which can be easily computed *via* an R Package (Pastore, 2018). Finally, a recent research study also proposes another effect size that can be easily understood by laypeople: The Persons as Effect Sizes (Grice et al., 2020), which describes the proportion of participants that match the specific theoretical expectation.

## Conclusion

Research studies in investigative interviewing and deception detection are a constantly growing field with important applied applications. Yet, improvements and the addition of less common methodological/statistical approaches might be needed. By applying them, it would be possible to have a more complete picture of the effectiveness of specific interviewing techniques and cues to deception. Furthermore, it would be possible to refine theories and to make the results more applicable to applied settings. In particular, it is important to implement person-centred approaches and to explore how different profiles moderate the effect on the outcome variable of several predictors such as interviewing strategy and veracity. This is not meant to say that the variable-centred approach should be disregarded. Rather, the researcher should select the approach according to the aims of the study. The person-centred approach can help cope with a central question in this area: Is *this* person lying? It will not be the ultimate solution, but it can help to increase specificity at the cost of a limited loss in parsimony.

Also, if we focus on the measures of the effect size other than the classical Cohen’s *d*, we can compare the efficacy of different techniques concerning not only mean differences between the experimental group but also their capability to discriminate group membership, such as truth telling vs. lying.

Two last points are worth reporting. First, although this research area has seen an increase in the reporting of Bayesian analyses, this is often limited to the reporting of Bayes factors. Yet, in doing so we are missing the most powerful tool of the Bayesian approach toolbox: cumulating and updating knowledge and evidence (Wagenmakers et al.,2018a,b). Future studies should hence try to implement all its features, including the use of prior and posterior distributions, whenever possible.

Second, it has already been suggested elsewhere that we need new measures for the testing of the efficacy of interviewing techniques. But apart from rare exceptions (Vrij et al., 2021), few new measures have been developed. A suggestion that we would like to provide is linked to the exploration of the efficacy of a specific technique to increase the elicitation of the information. The researcher could, for example, compare a given technique with another by focusing on the amount of useful and true information that is provided by the interviewee. Such measures could be obtained through a ratio between true information and false information (true information/true information + false information), which is similar to what is carried out in eyewitnesses research. The difference here is that false information should only account for deliberate lying (not for memory errors). This requires asking the participants what information is true and what information is false after the interview took place, but it would provide the researchers with interesting insight concerning how well a technique can maximise the amount of true information provided and, consequently, how well a technique discourages lying. Similarly, a ratio between relevant information and total information could be developed (relevant information/relevant information + irrelevant information), in collaboration with practitioners who could indicate what is relevant and what is not. Of course, such measures are just coarse-grained suggestions that need refining. We hope that this study will be useful for researchers and practitioners alike working in investigative interviewing.

## Author Contributions

NP conceived the idea of this article and wrote the manuscript. LC contributed to the writing of the article and provided feedback. Both authors contributed to the article and approved the submitted version.

## Conflict of Interest

The authors declare that the research was conducted in the absence of any commercial or financial relationships that could be construed as a potential conflict of interest.
